# Cecal volvulus as a rare complication of internal hernia after Roux-en-Y gastric bypass: a case report and literature review

**DOI:** 10.1093/jscr/rjae252

**Published:** 2024-04-24

**Authors:** Marvin Heck, Benjamin C Kensing, Hishaam N Ismael

**Affiliations:** Department of Surgery, University of Texas Health Science Center at Tyler, Tyler, TX 75708, United States; Department of Surgery, University of Texas Health Science Center at Tyler, Tyler, TX 75708, United States; Department of Surgery, University of Texas Health Science Center at Tyler, Tyler, TX 75708, United States

**Keywords:** cecal volvulus, Roux-en-Y gastric bypass, internal hernia, Petersen’s defect

## Abstract

This case report describes a rare instance of cecal volvulus resulting from an internal hernia through Petersen’s space, occurring 20 years after Roux-en-Y gastric bypass surgery, marking it as the second such case in English literature. An 81-year-old female presented with symptoms of bowel obstruction, and imaging findings concerning for cecal volvulus. Emergency surgery revealed necrotic bowel due to an internal hernia, necessitating a right hemicolectomy, with subsequent successful anastomosis and hernia defect closure. The incidence of internal hernias post-gastric bypass is notable, emphasizing the critical need for surgical vigilance. This case underscores the importance of considering internal hernias in differential diagnoses for bowel obstruction in post-bariatric surgery patients, highlighting the life-saving role of prompt surgical intervention in the management of cecal volvulus complications.

## Introduction

Cecal volvulus is a rare but potentially life-threatening condition characterized by torsion of the cecum around its mesentery, leading to bowel obstruction and ischemia. While cecal volvulus itself is an uncommon clinical entity, its occurrence as a complication of an internal hernia is even more unusual. This report presents the unique case of a cecal volvulus from an internal hernia of the terminal ileum, cecum and ascending colon via Petersons space after Roux-en-Y gastric bypass surgery 20 years prior, a commonly performed surgery for morbid obesity. To our knowledge, this is only the second case reported in the English literature [1].

## Case report

The patient is an 81-year-old female with a medical history significant for dilated cardiomyopathy, HFrEF, HTN, HLD, severe prosthetic aortic valve stenosis, who presented to the ED with diffuse abdominal pain, obstipation, and multiple episodes of emesis for ~24 h. Her surgical history included a Roux-en-Y gastric bypass 20 years ago (2003) complicated by small bowel obstruction requiring exploratory laparotomy in 2018.

Upon evaluation in the ED, she was a frail, ill appearing Caucasian female. Afebrile and hemodynamically stable with abdominal distension, diffuse tenderness, and voluntary guarding. On labs, the patient had a normal white count and lactate.

**Figure 1:**
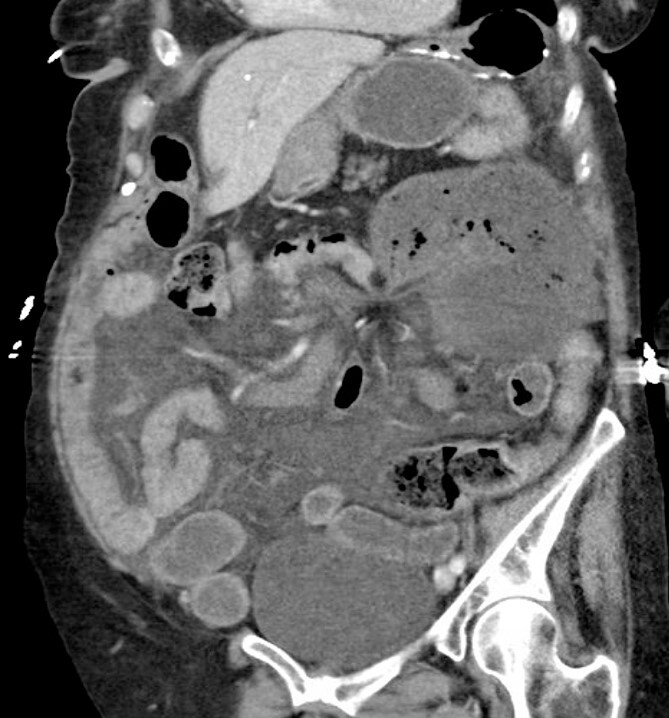
Computed tomography showing cecal volvulus secondary to internal hernia.

Computed tomography (CT) of her abdomen and pelvis with contrast revealed a dilated colon in the left upper quadrant with mesenteric edema and free fluid throughout the abdomen consistent with a cecal volvulus (Fig. 1).

**Figure 2:**
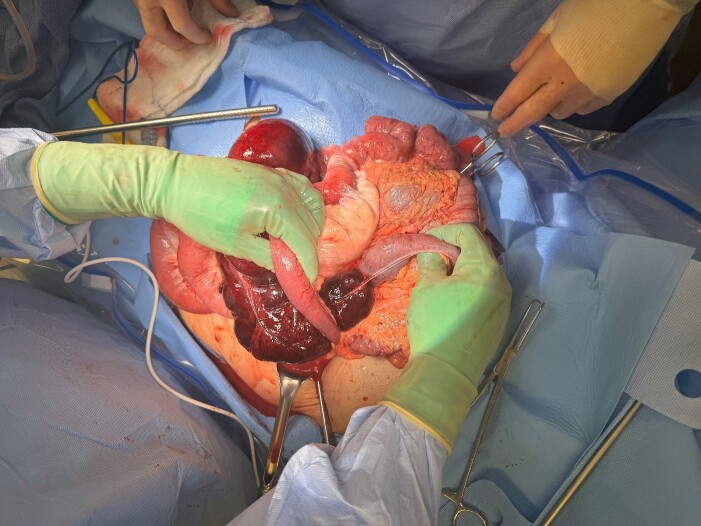
Cecal volvulus through Petersen’s defect.

The patient was taken for emergent exploration. Upon entry to the abdomen, a large amount of purulent drainage was encountered. The cecum and terminal ileum were in the left upper quadrant, and it became clear that this was an internal hernia through Petersen’s defect (Fig. 2). The right colon and terminal ileum were necrotic, the hernia was reduced, a right hemicolectomy was performed, the patient was left in discontinuity, and a temporary closure device was placed to perform a second look 24 hours after the index surgery. At her second surgery, a stapled ileocolic anastomosis was created, the Petersen’s defect was closed with an absorbable running suture, and the abdomen was closed. The patient recovered without complication and was discharged home on postoperative day 5.

## Discussion

Internal hernias following Roux-en-Y gastric bypass surgery represent a significant and potentially life-threatening complication. The cumulative incidence of internal hernia at 3 years post-laparoscopic roux-en-y gastric bypass has been shown to be around 5% in a recent retrospective analysis of a large database that included 46 918 patients [2]. This is similar to the reported incidence of 6.2% in other single institution and single surgeon case series [3].

The literature suggests that certain surgical techniques can influence the risk of developing an internal hernia. Antecolic Roux limb placement is generally associated with a lower incidence of internal hernia compared with the retrocolic approach [4, 5]. A recent large meta-analysis compared the incidence of internal hernias after Roux-en-Y gastric bypass surgery after mesenteric defect closure vs no closure and found a reduced incidence of 2% in patients who underwent defect closure vs 6% in non-closure [6]. A Swedish randomized controlled trial evaluated the incidence of major postoperative complications and small bowel obstruction within 3 years. The study showed that closure of the mesenteric defects leads to increased risk of early postoperative complications, mainly due to kinking of the jejunojejunostomy, but overall, significantly reduced the need for reoperation for small bowel obstruction over 3 years [4]. Despite this, no surgical technique has been proven to eliminate the risk of developing an internal hernia entirely. Patients most commonly present with internal hernias within the first 3 years after bariatric surgery secondary to intra-abdominal fat loss [2], especially if excess weight loss occurs rapidly, defined as greater than 90th percentile of expected weight loss [7].

Cecal volvulus in contrast to sigmoid volvulus is a surgical emergency due to low effectiveness of endoscopic detorsion and high risk of perforation, which can be attempted first in uncomplicated sigmoid volvulus. Colonoscopy does also evaluate the viability of the sigmoid colon. In the absence of colonic necrosis, detorsion can be attempted and semi-elective sigmoidectomy can be performed [8].

In this case, the patient presented with gangrenous bowel and required a right hemicolectomy. The patient was closed temporarily and underwent a second procedure due to the excessive amount of contamination encountered during the index operation. Detorsion and cecopexy without resection have been described for cecal volvulus but have a high risk of recurrence and are therefore not recommended [9].

## Conflict of interest statement

None declared.

## Funding

None declared.
